# Non-Hormonal Treatment Options for Regulation of Menstrual Cycle in Adolescents with PCOS

**DOI:** 10.3390/jcm12010067

**Published:** 2022-12-21

**Authors:** Elisabeth Reiser, Julia Lanbach, Bettina Böttcher, Bettina Toth

**Affiliations:** Department of Gynecological Endocrinology and Reproductive Medicine, Medical University of Innsbruck, 6020 Innsbruck, Austria

**Keywords:** polycystic ovary syndrome, oligomenorrhea, menstruation, metformin, GLP-1, menstrual cycle

## Abstract

Menstrual irregularities are one of the main clinical symptoms caused by polycystic ovary syndrome (PCOS). Pharmacological treatment options for non-fertility indications to restore menstrual frequency play an important role in the management of PCOS. Oral contraceptive pills are commonly prescribed for adolescents with menstrual irregularities, however, when contraindicated or poorly tolerated, further pharmacological therapy is required. This systematic literature research aims to provide an overview concerning the effects of non-hormonal pharmacological treatment options on menstrual irregularities in adolescents suffering from PCOS. A systematic literature search in PubMed, Cochrane, Embase, Bio-SISS and Web of Science was performed, including literature from January 1998 to September 2022, using specific keywords in order to find related studies. *n* = 265 studies were identified of which *n* = 164 were eligible for further evaluation. Only four placebo-controlled studies were identified, with diverging inclusion and exclusion criteria. Available data on specific non-hormonal off-label use medication primarily consisted of metformin, Glucagon-like peptide 1 receptor agonists, thiazolidinediones, anti-androgen agents (spironolactone, finasteride, flutamide) and supplements (chromium picolinate, myo-inositol). However, only a few have partly pointed out beneficial effects on improving menstrual frequency in patients diagnosed with PCOS. In summary, metformin in dosages of 1500–2550 g/day, GLP-1—analogues and supplements were effective in regulation of menstrual cycles in adolescents diagnosed with PCOS. Menstrual frequency in adolescents with PCOS is essential to prevent hypoestrogenism with long-term consequences. In this context, MET is the most effective and cost- efficient in overweight adolescent girls, also showing beneficial effects in the regulation of insulin sensitivity, especially if COCs are contraindicated or not well-tolerated. Further studies are needed to evaluate therapies in lean and normal-weight girls with PCOS.

## 1. Introduction

Polycystic ovary syndrome (PCOS) affects approximately 6 to 13% of adolescent girls [1,2]. Diagnosis and treatment remain a challenge due to the considerable heterogeneity in its manifestation and complexity of this health condition in adolescent young women [3,4]. Key features for diagnosing PCOS in adolescents include irregular menstruation and hyperandrogenism, in addition to specific diagnostic criteria for menstrual cycle irregularities since menarche was established [5]. The presence of polycystic ovary morphology by transvaginal ultrasound can also occur in adolescents not suffering from PCOS and is not specific [4,5].

Adolescents with PCOS mainly seek medical advice to alleviate burdensome PCOS-related clinical manifestations, such as acne, hirsutism, alopecia and oligo-/amenorrhea. To date, no pharmacological therapy has been approved by the FDA/EMA to treat clinical manifestations in adolescents suffering from PCOS [6]. According to a review by Vitek et al. [7], the current state of knowledge indicates that data obtained in several studies on a host of pharmacological substances suggest potential beneficial effects in terms of symptom relief.

According to the international evidence-based guideline for the assessment and management of PCOS published in 2018, therapeutic pharmacological options for non-infertility indications include combined oral contraceptive pills (COC), metformin (MET), anti-obesity agents, anti-androgen agents and Inositol, addressing different clinical manifestations [5]. Formerly, routine treatment focused on oral contraceptive pills. Within the last 20 years, treatment of adolescents with PCOS mainly focused on COC. Recently, adolescents in general tend to prefer non-hormonal contraceptive options to hormonal contraceptives [8].

This review focusses on non-hormonal treatment options for menstrual irregularities in adolescents with PCOS including MET, Glucagon-like peptide 1 receptor agonists, thiazolidinediones (rosiglitazone, pioglitazone), anti-androgen agents (spironolactone, finasteride, flutamide), combination treatment with SPIOMET and supplements (chromium picolinate, myo-inositol).

## 2. Methods and Materials

**Figure 1:**
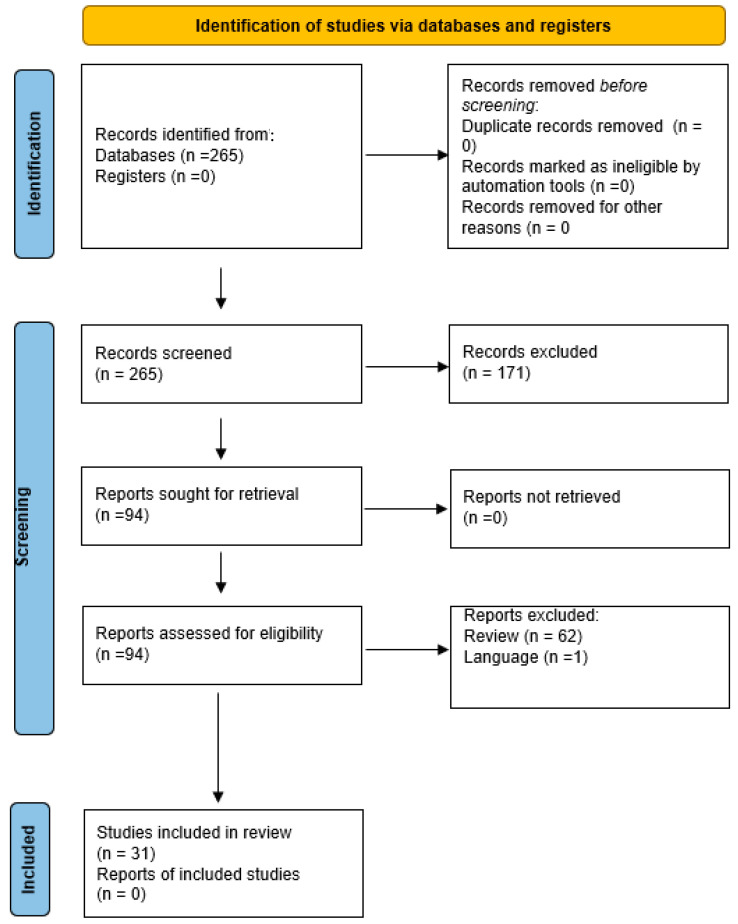
Flow diagram of the identification of studies via databases and registers.

The PubMed, Cochrane, Embase, Bio-SISS, and Web of Science databases, including studies, were searched from January 1998 to September 2022 by following the “preferred reporting items for systematic reviews and meta-analysis” (PRISMA). The following key words were used: oligomenorrhoea, adolescent, adolescence, PCOS, menstrual cycle, menstrual cyclicity, metformin, thiazolidinediones, spironolactone, flutamide glucagon-like peptide 1 receptor agonists, myo-inositol, herbal medicines. No abstracts or conference proceedings were included. Duplicates were removed and the remaining studies were screened by two independent authors. Selection process is displayed in Figure 1 (Flowchart). Information about yearly treatment costs were extracted from the Austria Codex, containing up-to-date professional information on all human and veterinary medicinal specialties approved in Austria. Some of the medication is covered by Austrian insurance, but since cost coverage differs between countries, the end user costs are listed.

## 3. Results

An overview of the analyzed studies and the therapeutic effects of Metformin, GLP-1- agonists, insulin sensitizers, anti-androgens, Myo-Inositol and supplements on menstrual irregularities in adolescents are presented in Table 1. An overview of treatment options, mode of action, indication, dose, side effects, safety during pregnancy, contraindications, and costs is demonstrated in Table 2.

### 3.1. Metformin

Metformin (MET) is the most commonly used drug to treat non-insulin dependent diabetes mellitus (type 2 diabetes, DM2), and the most thoroughly investigated insulin-lowering agent to treat PCOS. It is FDA approved for the treatment of DM2 in children aged 10 years and older but remains off-label use for the treatment of PCOS. The biguanide enhances insulin sensitivity in the liver by inhibiting hepatic glucose production and in the muscle by increasing glucose uptake and use [28].

A total of 10 studies investigating the effect of MET on menstrual irregularities in adolescents with PCOS and obesity were identified:

Two randomized controlled trials [9,10] investigated 36 obese adolescents (12–21 years) and 119 adolescents (mean age: 17.2 years) with PCOS who received either MET (1000 mg/d) or COC (ethinylestradiol 30 μg/norgestiate 0.25 mg) over 6 months or MET (1700 mg/d) vs. COC (30 μg ethinylestradiol and 15 mg progestin) vs. placebo over 24 months, respectively. The number of regular menstrual bleedings increased in both groups, but more in the COC groups (*p* = 0.007) and (92.5% vs. 100%).

When focusing on studies with MET alone, in obese adolescent girls 1700 mg MET/day led to regular menstrual cycles already after the first month of treatment and continued up to six months after the end of treatment [11]. This was also evident in the studies of Glueck et al. [13,14,15] assessing changes in menstrual frequency over a study period of at least six months. The studies included 11–20 obese girls (14–18.9 year) with PCOS undergoing MET (1500–2550 g/d) therapy for 9 up to 12 months and all showed a significant improvement in menstrual regularities during MET therapy.

The influence on menstrual frequency of lifestyle modification ± MET treatment (2000 mg/d) in 22 obese adolescents with PCOS over a six months period was investigated in a pilot randomized double-blind study [17]. No difference in the number of menstrual bleeding episodes in the MET group compared with the placebo group were found. In non-obese girls (aged 13–20 years) receiving 1275 mg MET/d for six months regular menstrual cycles were reported within 4 months [18].

Another study consisted of a single and a combination treatment trial [12]. MET (1700 mg/d) was compared to COC (30 μg ethinyl estradiol (EE) and 0.15 mg desogestrel) and lifestyle modification and placebo in 79 obese adolescent girls (12–18 years) for 24 weeks. When looking into the groups without COC, the following number of cycles were observed: 3.2 under MET vs. 2.3 under lifestyle modification vs. 2.5 under placebo per 24 weeks without significant difference between the groups.

When even higher MET doses (2000 mg/d) were applied in obese adolescent girls [16] and compared to COC (30 μg EE, 1 mg norethindrone acetate (NETA)) the number of menstrual cycles was also higher in the COC group.

To sum up, MET in adolescent obese girls with PCOS was beneficial in achieving regular menstrual cycles with dosage from 850–2550 mg/d. Of note, in three studies PCOS diagnosis included sonographic presences of PCO-like ovaries, which is no longer applicable in the first 8 years after menarche [5]. Side effects mainly consisted of nausea and/or diarrhea. Yearly costs for MET 1500 mg/d are 58.40 Euro. Patients need to be informed about the off-label use.

### 3.2. Glucagon-like Peptide 1 Receptor Agonists

Glucagon-like peptide 1 receptor agonists (GLP-1RA) share similar glucoregulatory and central characteristics of the endogenous gut hormone GLP-1, a member of incretin hormones secreted in the intestinal tract in order to enhance postprandial insulin secretion [31]. Due to these insulinotropic effects, GLP-1 reduces hepatic glucagon release, delays gastric emptying, slows down intestinal motility, improves glycemic control, stimulates the hypothalamic satiety center and induces weight loss as a result of suppressing appetite [32].

The clinical effectiveness of GLP-1RA with regard to the treatment of DM2 and weight management in people suffering from obesity (BMI >30 kg/m^2^ or >27 kg/m^2^ with at least one weight-related coexisting condition, such as dysglycemia, hypertension, dyslipidemia or obstructive sleep apnea) led to their approval by the FDA and EMA for the indications mentioned above [37,38].

Owing to the fact that PCOS is often associated with impaired glucose tolerance and higher rates of obesity, GLP-1RA, Liraglutide (LIRA, a long-acting GLP-1RA) and Exenatide (EX, a short-acting GLP-1RA) in particular, have recently been considered to be a prospective therapeutic option for the management of women with PCOS and obesity [39].

To our knowledge, no clinical trials are currently available which assess the effects of GLP-1RA on menstrual regularity in obese adolescents with PCOS.

Ornstein et al. (2011) found that there is evidence that weight loss achieved through diet management and lifestyle intervention can help restore menstrual regularity in obese adolescents with PCOS (12–22 years) [40].

Due to the lack of studies assessing the effects of GLP-1RA in adolescents with PCOS on menstrual irregularities and the great heterogeneity concerning dosage, the length of treatment and outcome in adult women, studies are required to determine benefits and safety issues before the treatment with GLP-1RA can be advised. Common side effects of GLP-1RA reported in the vast majority of trials were gastrointestinal in nature. Another important aspect which has to be taken into consideration are the high treatment costs of LIRA and EX, amounting to a considerable sum of €25,301.8 with LIRA and €91,417.9 with EX per year.

### 3.3. Thiazolidinediones: Rosiglitazone and Pioglitazone

Thiazolidinediones (TZDs) are insulin sensitizers regulating energy homeostasis, glucose and lipid metabolism mainly by acting as an agonist to the peroxisome proliferator-activated receptor (PPAR)-γ. Two TZDs, rosiglitazone (ROS) and pioglitazone, have been approved as monotherapy and combination therapy for the treatment of type 2 diabetes [33].

Due to their beneficial effects on function in glycemic control and improvement of insulin resistance, TZDs have been considered a therapeutic option for the management of patients with PCOS [41].

Limited RCTs are available which assess the effectiveness of TZDs on controlling menstrual irregularity in adolescents with PCOS.

Two studies evaluated pioglitazone 30 mg/d for 6 months in 22 respective 15 young obese women (15–24 years and 19.4 ± 3.8 years) with PCOS and found a significant enhancement of menstrual frequency (*p* < 0.0001 [20]) [20,21].

A randomized double-blind parallel clinical trial compared treatment with COC (30 µg EE, 3 mg drospirenone) and ROS (4 mg/d) in adolescent obese girls (10–20 years) with PCOS over a period of 6 months. No statistically significant results were found when analyzing changes in menstrual patterns [22].

Concerns about safety issues and side effects, including weight gain, edema and risk potential in pregnancy, limited the use of TZDs for PCOS among young women without diabetes. However, given small sample sizes and the lack of sufficient evidence-based supports, further studies are required to give recommendations in young women with PCOS.

The costs of treatment with pioglitazone and rosiglitazone amount to €164.25 per year, respectively.

### 3.4. Antiandrogens Flutamide, Finasteride, Spironolactone and SPIOMET

Elevated androgen levels which clinically manifest as hirsutism, acne and/or alopecia are often associated with high levels of suffering among adolescents with PCOS. For that reason, antiandrogen agents have been considered an essential therapeutic strategy to lower androgen excess and thus attenuate their clinical manifestations. Two types of antiandrogens have been proposed for the management of PCOS: antagonists to the androgen receptor, such as spironolactone and flutamide and inhibitors of 5-alpha reductase, such as finasteride [42]. However, there is limited data on the clinical effects of treatment with antiandrogens and combined therapy in adolescents with PCOS.

### 3.5. Flutamide

Flutamide, a nonsteroidal antiandrogen drug, acts as a selective antagonist to the androgen receptor. Therefore, androgen uptake and the capability of androgens binding to target tissues are inhibited [34].

Several studies have proved the beneficial effects of flutamide on reducing hirsutism [43]. Advantageous effects of flutamide on ovulation rates and menstrual frequency have been reported although data on this use are more conflicting.

A clinical trial including eight adolescent girls (16–19 years) with PCOS suffering from moderate to severe hirsutism (Ferriman and Gallwey score mean score of 16.4 ± 3.2) and irregular menstruation evaluated the effect of flutamide (250 mg/2× d for 6 months) on hormonal levels, menstrual regulation and ovulation rates. Cycle regulation was gained in all participants [23].

In another clinical trial of 18 non-obese adolescents (14–18 years) with PCOS and a history of precocious pubarche no significant changes were observed regarding menstrual frequency with flutamide 250 mg/d for 18 months [24].

One of the major concerns when prescribing flutamide are the potentially severe harming side effects of flutamide on liver function which have been reported in adult and adolescent women [34]. Nevertheless, hepatotoxicity can be avoided and beneficial effects of flutamide can be obtained if low doses (<250 mg/d) are administered [44].

### 3.6. Finasteride

Pharmacological therapy with finasteride results in a suppressed production of dihydrotestosterone (DHT) as it acts as an inhibitor of steroid type II 5-alpha-reductase, an enzyme responsible for the biosynthesis of DHT [35]. Treatment with intermittent low-dose finasteride in adolescents with PCOS showed beneficial effects on hirsutism scores [45].

This research revealed that no clinical trials on regulation of the menstrual cycle among adolescents with PCOS have been conducted so far.

### 3.7. Spironolactone

Spironolactone (SPIRO), an aldosterone antagonist and diuretic with antiandrogen properties, is commonly used in adolescents with PCOS to treat elevated androgen levels and their clinical manifestations [6].

An open-label randomized study compared treatment with MET (1000 mg/d) and SPIRO (50 mg/d) for 6 months and its effects on regulating menstrual cycle involving 69 adolescents (MET *n* = 35, SPIRO *n* = 34) (22.6 ± 5.0 years) with PCOS. Both, MET and SPIRO helped to restore regular menstrual cycles, which was more significant in the SPIRO group [25].

The most common side effects of SPIRO are mostly mild and dose-dependent, including metrorrhagia, abdominal pain, dryness of the mouth and polyuria [25].

### 3.8. SPIOMET

Ibáñez et al. [26] analyzed two open-label controlled randomized pilot studies in which they compared treatment with a COC (20 μg EE, 100 mg levonorgestrel) with a low-dose combination (SPIOMET) of SPIRO (50 mg/d), pioglitazone (7.5 mg/day) and MET (850 mg/d) for 1 year in 62 non-obese and non-diabetic adolescent girls (mean age 15.8 years) with PCOS. In the post-treatment year menstrual cycles remained more regular in 90% of the adolescents treated with SPIOMET and 42% of adolescents treated with OC.

The costs of treatment with spironolactone and flutamide amount to €315.4 and €262.8 per year, respectively.

Due to lack of data and high-quality RCTs with sufficient sample sizes investigating the effects of antiandrogens on regulation of menstrual cycles, further studies are needed before treatment options with antiandrogens in adolescents with PCOS can be recommended.

### 3.9. Supplements

Additional supportive therapies to alleviate symptoms caused by PCOS include herbal medicines and supplements. However, only preliminary data on efficacy and safety of complementary medicine is available. Several herbal medicines and supplements have been proposed that may exert favorable effects on clinical symptoms caused by PCOS [46]. In our research, the focus was on the following supplements: fenugreek seed extract and chromium (III). Most of the studies were performed on adult women, especially with herbal medicines such as *Vitex agnus-castus* L. (chaste tree, chasteberry) but only data on adolescents with PCOS were reflected.

Chromium (III) combined with picolinic acid to enhance intestinal absorption, plays an important role in regulating glucose and insulin homeostasis. Data on treatment with chromium (III) picolinate suggested positive effects on PCOS in adult women [36]. Amr et al. [27] evaluated the effects of chromium (III) picolinate (1000 µg/d) in 35 adolescents (<18 years) diagnosed with PCOS for 6 months. At the beginning of the clinical trial 82.9% of the adolescents suffered from oligo-/amenorrhea. After 6 months of therapy the number of patients experiencing oligo-/amenorrhea decreased significantly (83% before vs. 31% after 6 months of treatment) (*p* < 0.001).

Considering the aforementioned clinical studies, supplements provide preliminary evidence that these substances may exert promising effects on improving clinical symptoms, oligo-/amenorrhea in particular, caused by PCOS. However, further studies, especially in adolescents, are needed that may corroborate the positive effects on restoring menstrual cycles.

Cost of chromium (III) picolinate is less than €30 per year.

### 3.10. Myo-Inositol

Myo-inositol (MYO) belongs to the vitamin B complex group. In women with PCOS, reduced MYO levels are associated with insulin resistance [47]. In PCOS patients with hyperinsulinemia the D-chiro-inositol (DCI)/MYO is increased (by a reduction of MYO), leading to an overproduction of androgens in the ovary [48,49,50]. MYO administration can lead to the following improvements: regular menstrual cycles, ovulation, reduced testosterone levels, and reduced insulin resistance in adult women with PCOS [29,30].

Only one study reported on the influence of MYO on menstrual cyclicity in adolescent girls with PCOS:

In the study by Cirillo et al. [19], 23 adolescent PCOS patients (mean age: 17.22 ± 0.72) assigned to treatment with an association of 400 mg Alpha-Lipoic Acid (ALA) and 1000 mg MYO (2×/d) for 3 months and once daily for further 3 months were compared to 21 matched healthy controls. Menstrual frequency was improved in the PCOS patients as follows: Five oligomenorrheic patients presented regular cycles, and four amenorrhoeic adolescents developed oligomenorrhoea.

MYO is currently considered an experimental therapy in PCOS by the ESHRE guideline [5]. Yearly costs are 197.10 Euro for the treatment with 1000 mg/day.

## 4. Discussion

Given the fact that PCOS is a lifelong condition patients have to cope with, pharmacological treatment options targeting specific clinical manifestations of PCOS are required. Menstrual disorders, oligo- and amenorrhea in particular, are one of the main clinical manifestations in PCOS, primarily treated with COCs. However, not all patients benefit from COCs due to side effects and adverse reactions caused by hormonal therapy. In order to increase the number of therapy options in adolescents suffering from PCOS, the effects on menstrual frequency of various off-label use medications approved for other indications have been investigated. There is evidence that enhancing insulin sensitivity is associated with improved menstrual frequency, which suggests that medication targeting glucose metabolism and weight control exerts beneficial effects on regulation of the menstrual cycle, especially in overweight women [51]. Metformin shows an overall benefit, while GLP-1RA and ROS show conflicting data on improvement of menstrual frequency or were only studied in adult women. The meta-analysis by Al Khalifah et al. [42] showed no statistically important difference among the interventions (metformin, oral contraceptives, placebo, pioglitazone, spironolactone, flutamide, and lifestyle interventions) in improvement of menstrual cycles in adult women.

Several studies investigating the effect of non-hormonal treatment of women with PCOS are currently conducted as displayed at clinicaltrials.gov as of 13 December 2022 (Metformin: 178, Myo-Inositol 43, Glucagon-like peptide receptor agonists: 20, Thiazolidinediones: 3, Spironolactone: 11, Flutamide: 6, Chromium picolinate: 2, cinnamon: 4). However, only 11 studies focus on adolescent PCOS patients. This low number can certainly be explained by the stricter requirements and difficult feasibility of studies in minors. Nevertheless, the increasing number of overweight adolescents at risk under COC strengthen the justification of conducting clinical trials in this cohort [42]. Especially therapeutic options with GLP-1-agonists in adolescents with PCOS seem promising. Due to the lack of studies in adolescents with PCOS, results of studies in adult women could be considered to evaluate new options for girls:

An improvement of menstrual irregularities by the GLP-1- agonist liraglutid was found in several studies: Liraglutid treatment resulted in a significantly higher frequency of menstrual bleedings compared to placebo with no change in the frequency of menstrual bleeding [52]. Similar results were found in a 26-week placebo-controlled trial: 72 overweight women with PCOS were assigned to receive either liraglutid or placebo in a 2:1 ratio. Significant results were achieved in the liraglutid group with regard to menstrual frequency [53]. On the other hand, in the following studies no superiority of either metformin or liraglutid was found: No statistically significant results were reached when analyzing changes in menstrual patterns in obese women with PCOS in any of the three treatment arms metformin, liraglutid or the combination of both over 12 weeks [54]. Assumingly, the duration of treatment was too short to notice significant effects. Another study, also conducted over 12 weeks, showed similar results as no significant changes in menstrual frequency between metformin and liraglutide treatment were found [55].

Further long-term studies with GLP-1-agonists are needed to evaluate the impact on PCOS symptoms. Possibly, promising results might open new prospects of assumption of cost by health insurances as at the moment GLP-1-agonists are only paid by the insurance in case of metformin resistance in DM Typ2 patients.

Treatment of hyperandrogenism with antiandrogen medication is mainly recommended to ameliorate clinical manifestations of androgen excess (e.g., hirsutism and acne). The combination of metformin and spironolactone showed superior effects on menstrual frequency compared to monotherapy with either metformin or spironolactone over six months [56]. However, the role of antiandrogen agents to improve menstrual frequency remains controversial [5]. Importantly, in order to avoid possible feminizing effects on genital development of male fetuses, effective contraception should be part of the counselling and be prescribed along with antiandrogenic medication whenever treating sexually active adolescents [6]. Early initiation of antiandrogen treatment in adolescents may even have an impact on the incidence of childbirth after spontaneous conception in adulthood. The period of time to first childbirth after spontaneous conception was reduced in women who received antiandrogenic medication earlier during adolescence compared to women who were treated with antiandrogenic medication after adolescence [57].

An important treatment option, which has not been discussed so far, as no data for adolescents exist, consists of phytotherapeutic extracts of the *Vitex agnus-castus* L. fruit. It is commonly prescribed to alleviate a range of gynecological disorders, such as premenstrual syndrome (PMS), abnormal uterine bleeding, hyperprolactinemia, luteal phase defect and premenstrual mastodynia [58]. A randomized triple-blind placebo-controlled clinical trial compared treatment with low-dose COC, *Vitex agnus-castus* L. and the impact on regularization of menstrual cyclicity for three months in women with PCOS. Treatment with *Vitex agnus-castus* L. showed similar beneficial effects on regulating menstrual frequency compared to low-dose oral contraceptives [59]. As *Vitex agnus-castus* L. is well-tolerated, research of the effects in adolescent girls and the approval for this age group could be promising.

Combined therapy options addressing various manifestations have shown promising results in symptom management in PCOS patients, but data are still limited. The additives of combined therapies vary widely, therefore conclusions are difficult to draw.

Data on additional supportive therapy options including herbal medicines and supplements suggest a benefit on regulation of the menstrual cycle. Unfortunately, no placebo-controlled trials in adolescents were published so far. In adult women with PCOS, a review of 33 studies with herbal medicines showed beneficial effects on menstrual cycles and hyperandrogenism in women with PCOS but especially pre-clinical studies to explain the effects of herbal medicines are needed [60].

This is the first review solely focusing on non-hormonal treatment of menstrual irregularities in adolescent PCOS patients. The exclusion of a rather large number of studies due to a lack of data in adolescents can be considered as a possible limitation. Criteria for PCOS in adolescents have only been defined in 2017/2018, so all studies before that date use inconsistent definitions which complicates the comparison of studies. Furthermore, studies with adolescents may include very limited numbers of participants. The fact that a large number of girls decide on hormonal contraceptives hinders the evaluation of non- hormonal treatment options.

One main aspect remains cost-benefit efficacy as it has to be taken into consideration when prescribing medical agents. Yearly treatment costs of several ten thousands of Euro in case of GLP-1RA have to be compared to 58 Euro for MET or similar costs for COC.

Effectiveness and safety issues were not fully evaluated in adolescents, highlighting the need for further high-quality research on pharmacological treatment in adolescents suffering from PCOS.

## 5. Conclusions

Adolescents with PCOS mainly seek their gynecologist due to menstrual irregularities. Oligo- and Amenorrhea lead to hypoestrogenism with long- term consequences. MET seems to be an effective treatment option in overweight adolescent girls with PCOS. Supplements, such as MYO and Chromium (III) picolinate, and anti- androgens, such as spironolactone, also in combination with MET and piogliatazone, might be efficient alternatives. Further studies are needed to evaluate treatment options especially in lean and normal-weight adolescent girls with PCOS.

## Figures and Tables

**Table 1:** Included Literature. (Abbreviations are explained below).

Author, Year	PCOS Criteria	Age (Years)	BMI (kg/m^2^)	Intervention	Sample Size (Trial/ Control Group)	Length (Months (mo) or Weeks (we))	Outcome (Menstrual Cyclicity)	Other Outcomes
Allen, 2005 [9]	clinical/biochemical hyperandrogenism and oligomenorrhoea	12–21	40.1 ± 2.1 (COC) 37.3 ± 1.3 (MET)	MET 1000 mg/d COC: 30 μg EE and norgestiate 0.25 mg	16/15	6 mo	Regular menstrual cycle in both groups	Weight/BMI ↓ * Acne score ↓ * Testosterone ↓ * Hirsutism ↓ *
El Maghraby, 2015 [10]	Rotterdam criteria	17.2 ± 2.0	-	MET 1700 mg/d COC: 30 µg EE and 15 mg progestin Placebo	40/40/39	24 mo	Regular menstrual cycle in MET/COC group	Weight/BMI (only MET group) ↓ * Hirsutism ↓ *
De Leo, 2006 [11]	Rotterdam criteria	15–18	25.5–27.0	MET 1700 mg/d	18	12 mo	Regular menstrual cycle	Weight/BMI ↓ Androgen levels ↓ Hirsutism ↓ *
Hoeger, 2008 [12]	Menstrual irregularity (<eight menses in the preceding year) and clinical or biochemical evidence of hyperandrogenism	12–18	34.3–37.8	MET 1700 mg/d COC: 30 μg EE and desogestrel 0.15 mg lifestyle modification placebo	16/21/21/19	8 mo	Regular menstrual cycle in COC group/no difference in other groups MET group 75% of cycles with ovulation	Weight/BMI ↓ *
Glueck, 2001 [13]	Oligo-/amenorrhoea + clinical or biochemical evidence of hyperandrogenism	14–18.9	33.6	MET 1500–2550 g/d	11	9 mo	91% regular menstrual cycle during MET	Weight ↓
Glueck, 2006 [14]	Rotterdam criteria	<20	30.8	MET 1500–2550 g/d + diet	35	12 mo	74% with regular menstrual cycle	Weight ↓ * Cholesterol ↓ * Triglyceride ↓ * HOMA↓ * Testosterone ↓ *
Glueck, 2009 [15]	Rotterdam criteria	14–17	30.4	MET 1500–2550 g/d + diet	20	12 mo	82% with regular menstrual cycle	Weight/BMI ↓ * Testosterone ↓ * Cholesterol ↓ * Triglyceride ↓ * HOMA↓ *
Al-Zubeidi and Klein, 2014 [16]	NIH criteria	14–18	33.7	MET 2000 mg/d COC: 30 μg EE and 1 mg norethindrone acetate	10/12	6 mo	Number of cycles higher in COC group	Androgen (only COC group) ↓ * BMI/ weight ↓ * HOMA ↓
Ladson, 2010 [17]	NIH/NICHD criteria	16.1 ± 1.5	35.9–37.1	MET 2000 mg/d ± lifestyle modification	22	6 mo	No difference	Acne score ↓ *
Ibáñez, 2000 [18]	Hirsutism and/or biochemical hyperandrogenemia, and oligoamenorrhea + precocious pubarche	13–20	21.9 ± 0.9	MET 1275 mg/d	8	6 mo	Regular menstrual in all patients, irregular within 3 months after withdrawal	Hirsutism ↓ * Testosterone levels ↓ *
Cirillo, 2019 [19]	Rotterdam criteria	17.2 ± 0.7	-	Alpha-Lipoic Acid (ALA) 400 mg and MYO 1000 mg/2×/d placebo	23/21	3 mo	Improved menstrual frequency	hirsutism ↔ acne ↔
Narsing, 2009 [20]	Clinical features of PCOS (Chronically anovulating, oligo-/amenorrhea, hyperandrogenism)	15–25	29.5 ± 7.9	Pioglitazone 30 mg/d	22	6 mo	Improved menstrual frequency (91% with regular cycles at the end of therapy)	Body weight/BMI ↔ Insulin resistance ↓ *
Stabile, 2014 [21]	Rotterdam criteria	19.4 ± 3.8	25.2 ± 5.1	Pioglitazone 30 mg/d	15/15	6 mo	Improved menstrual frequency	Body weight ↑ * Hirsutism and acne ↓ * Insulin resistance ↓ *
Tfayli, 2011 [22]	NIH criteria	10–20	35.6 ± 1.5	COC: 30 μg EE and drospirenone 3 mg ROS 4 mg/d	23/23	6 mo	No changes in menstrual cyclicity	Body weight/BMI ↔ glucose tolerance status ↔ visceral adiposity ↓ * (With rosiglitazone)
De Leo, 1998 [23]	Clinical diagnosis of PCOS based on hyperandrogenism, chronic anovulation, polycystic ovaries by ultrasound	16–19	-	Flutamide 500 mg/d	8	6 mo	Improved menstrual frequency	Hirsutism ↓ * Androgen levels↓ * Ovulatory cycles ↑ * Ovarian volume ↓ *
Ibáñez, 2000 [24]	Clinical features of PCOS (oligo-/amenorrhea, hyperandrogenism)	16.8 ± 0.3	-	Flutamide 250 mg/d	18	18 mo	No changes in menstrual cyclicity	Hirsutism ↓ * BMI ↔
Ganie, 2004 [25]	NIH criteria	22.6 ± 5.0	26.8 ± 4.0 (SPIRO) 26.5 ± 5.6 (MET)	MET 1000 mg/d SPIRO 50 mg/d	35/34	6 mo	Improved menstrual frequency	Hirsutism ↓ * Serum Testosteron Levels ↓ *
Ibáñez, 2020 [26]	Clinical diagnosis of PCOS based on hirsutism (score > 8 on modified Ferriman-Gallwey scale) and oligomenorrhea (menstrual intervals >45 days)	15.9 ± 0.2 (EE and Levonorgestrel) 15.7 ± 0.2 (SPIOMET)	24.9 ± 0.8 (EE and Levonorgestrel) 24.2 ± 0.7 (SPIOMET)	COC: 20 μg EE and 100 mg levonorgestrel SPIOMET: spironolactone 50 mg/d, pioglitazone 7.5 mg/d and metformin 850 mg/d	31/31	12 mo of treatment and 12 mo follow-up without treatment	Improved menstrual frequency (with SPIOMET even in the follow-up year)	Ovulation rates ↑ * (with SPIOMET) Hepatic-visceral-fat-excess ↓ * (with SPIOMET) HOMA-IR ↓ * (with SPIOMET)
Amr, 2005 [27]	Rotterdam criteria	14–17	BMI SDS 1.9 ± 0.7	Chromium (III) picolinate 1000 µg/d	35	6 mo	Improved menstrual frequency	Acne and hirsutism ↔ Free testosterone levels ↓ *

* = statistically significant, ↓ = reduction., ↔ = stable, ↑ = increase.

**Table 2:** Overview of treatment options, mode of action, indication, dose, side effects, safety during pregnancy, contraindications, and costs. (Abbreviations are explained below).

Medication	Mechanism(s) of Action	Off-Label Reproductive Use	Dosage	Main Side Effects	Teratogenic Effects (FDA Pregnancy Category)	Contraindications	Treatment Costs (per Year)
Treatment	Mode of Action	Indication	Dose	Side Effects	Safety during Pregnancy	Contraindications	Costs
Metformin [28]	Enhancement of insulin sensitivity Inhibition of hepatic glucose production Increased glucose uptake in the muscle Hyperandrogenemia ↓	Improvement of ovulation rates, menstrual regulation, hirsutism, weight loss	850–2550 mg/d	Nausea/vomiting/abdominal pain/ diarrhea	Pregnancy category B	Severe liver/kidney/heart insufficiency	58.4 Euro (1500 mg/d)
Myo-Inositol [29,30]	Increased MYO levels improve D-chiro-inositol/MYO ratio and reduce androgen production	Nutritional supplement	2000 mg/d	No	Unknown	No data available	197.1 Euro (2000 mg/d)
Glucagon-like peptide receptor agonists: exenatide, liraglutide [31,32]	Insulinotropic effects: hepatic glucagon release ↓, delays gastric emptying, intestinal motility ↓, glycemic control ↑, stimulates the hypothalamic satiety center, appetite↓, weight loss	Weight loss, menstrual regulation	Liraglutide: 1.2–3 mg/d Exenatide: 20 µg/d	Nausea/vomiting/abdominal pain/ diarrhea/injection site reaction/headache	Pregnancy category C	History of medullary thyroid carcinoma/ MEN 2/pancreatitis/renal impairment	LIRA 25,301.8 Euro (3 mg/d) Exenatide 91,417.9 Euro (20 µg/d)
Thiazolidinediones: rosiglitazone, pioglitazone [33]	PPAR-g receptor agonist: enhances cellular responsiveness to insulin, insulin-dependent glucose disposal ↑, glycaemic control ↑	Improvement of ovulation rates, menstrual regulation	Pioglitazone: 30 mg/d rosiglitazone: 4–8 mg/d	Weight gain/ abnormal vision, respiratory infection, numbness	Pregnancy category C	Heart failure/liver failure/bladder cancer (pioglitazone)	Pioglitazone 164.25 Euro (30 mg/d)
Flutamide [34]	Competitively binds androgen receptors → inhibits androgen uptake and/or nuclear binding of androgen	Treatment of signs of hyperandrogenism (e.g., acne, hirsutism)	<250 mg/d	Breast swelling or tenderness/nausea/vomiting/abnormal liver function	Pregnancy category D	Liver and kidney problems/ heart disease	262,8 Euro (250 mg/d)
Finasteride [35]	Inhibitor of 5-alpha reductase → antiandrogenic effects	Treatment of signs of hyperandrogenism (hirsutism)	2.5–5 mg/d	Reduced libido/ depression/ headaches/ gastrointestinal disorders	Pregnancy category X	Pregnancy/ liver disease/	153.3 Euro (5 mg/d)
Spironolactone [25]	Aldosterone antagonist, diuretic with antiandrogen properties	Treatment of signs of hyperandrogenism (e.g., acne, hirsutism)	50 mg/d	Gastrointestinal problems/ headache/ tenderness of the breasts/ menstrual disorders/dizziness	Pregnancy category C	Hyperkalemia/ chronic adrenal insufficiency	315.35 Euro (50 mg/d)
Chromium (III) picolinate [36]	The exact mechanism of action is not known → improved insulin sensitivity	Menstrual regulation and ovulation rates	200–1000 µg/d	No data available	Unknown	No data available	30 Euro

↑ = increase, ↓ = decrease and → = leading to.

## Abbreviations

BMI	Body Mass Index
COC	Combined oral contraceptives
d	per day
FDA	Food and Drug Administration
HOMA-IR	Homeostasis Model Assessment-Insulin Resistance
LIRA	Liraglutid
MEN	Multiple Endocrine Neoplasia
MET	Metformin
Mo	months
MYO	Myoinositol
NICHD	National Institute of Child Health and Human Development
NIH	National Institute of Health
PCOS	Polycystic Ovary Syndrome
PPAR	Peroxisome Proliferator activated receptor
ROS	Rosiglitazone
SDS	standard deviation score
SPIOMET	Spironolactone, pioglitazone, metformin
SPIRO	Spironolactone
we	weeks
